# The Bank of Japan’s exchange traded fund purchases: a help or hindrance to market efficiency?

**DOI:** 10.1057/s41260-023-00307-2

**Published:** 2023-03-21

**Authors:** Ailie Charteris, Conrad Alexander Steyn

**Affiliations:** University of Cape Town, Cape Town, South Africa

**Keywords:** Exchange traded funds, Market efficiency, Long-range dependence, Price delay, Monetary policy, Asset purchase programmes, E52, E58, G10, G14

## Abstract

We examine the impact of the Bank of Japan’s exchange traded fund (ETF) purchases on two aspects of market efficiency—long-range dependence and price delay—of the TOPIX and Nikkei 225 indices. An increase in ETF purchases results in lower long-range dependence for both indices while the impact on the price delay varies according to index and measure. A sub-period analysis shows that the impact on market efficiency varies over time, with the dominant pattern being a delayed harmful effect, followed by a positive impact and thereafter a negative effect. The implications of these findings are discussed.

## Introduction

As part of their monetary policy, the Bank of Japan (BOJ) began purchasing exchange traded funds (ETFs) tracking the TOPIX and Nikkei 225 indices in December 2010. While the purchasing programme was only intended to last for a year with a limit of ¥450 billion, it continues more than a decade later, with the purchase limit extended eight times with an annual limit of ¥12 trillion.^1^ However, ETF purchases in 2021 were the lowest since the introduction of the policy. As of 30 September 2021, the BOJ owns more than ¥36.4 trillion in ETFs, which equates to approximately 80% of all Japanese ETFs and 7% of the value of the first section of the Tokyo Stock Exchange (TSE) (Fujikawa 2021; Lee and Fujioka 2022). The BOJ is now the largest shareholder on the TSE, overtaking the Japanese Government Pension Fund. Since 2011, Japanese ETF assets have expanded by 2222% from ¥2.7 to ¥60 trillion (Lee and Fujioka 2022).

In an environment of low interest rates, many central banks turn to large scale asset purchase programmes (quantitative easing) to increase money supply, typically purchasing government bonds (Petrov 2017). While some central banks purchase private domestic securities such as corporate bonds, stock market purchases are rare and, if any, are limited to foreign securities as part of foreign exchange reserves.^2^ Although the BOJ’s holdings of ETFs are small in comparison to its holdings of government securities (¥528 trillion as of 30 September 2021), their involvement in the domestic stock market is unprecedented. Such a strategy is risky because ETFs do not mature like bonds meaning that at some point this policy must be unwound. Since the introduction of the purchasing policy, the BOJ has not sold any ETFs (Lee and Fujioka 2022).^3^ The purpose of the BOJ’s ETF purchases has been to encourage risk taking by reducing asset risk premiums, lowering the cost of capital for companies, and indirectly boosting economic activity in an environment of persistently low or negative inflation (Petrov 2017; Harada and Okimoto 2021). According to Hattori and Yoshida (2020), this policy was one of the BOJ’s last resorts to prevent market turmoil after the Global Financial Crisis in 2008, having exhausted all other monetary policy instruments.

An ETF is a basket of stocks that tracks an index. Shares in ETFs are traded on an exchange, but if there is excess demand for an ETF, the component stocks can be purchased^4^ and delivered to the fund manager who creates shares in the ETF (the opposite is true if there is excess supply of ETF shares) (Charteris 2013). Purchases of the constituent stocks drive their prices upwards. The price of the ETF should track the net asset value (NAV) closely, otherwise arbitrage opportunities arise. If the NAV is below the price, market participants can purchase the underlying securities and exchange them for shares in the ETF which can then be sold at the market price (the opposite is true if the NAV is above the price). Accordingly, if demand for the ETF drives the price of the ETF above its NAV, this will lead to greater demand for the constituent stocks, contributing to their prices rising (Da and Shive 2018). Theoretically, therefore, substantial purchases of ETFs by the BOJ will contribute to price increases of the underlying stocks. Barbon and Gianinazzi (2019) and Harada and Okimoto (2021) confirm that the prices of the stocks contained in the ETFs rise following purchases by the BOJ.

According to the Efficient Market Hypothesis (EMH), an efficient market is one in which share prices fully reflect all available information (Fama 1970). The Adaptive Markets Hypothesis (AMH) of Lo (2004) suggests that market efficiency is not a static but rather a dynamic concept, meaning that there are periods when prices reflect all available information and periods when they do not. For prices to be efficient, it is necessary for investors to analyse stocks in order to identify mispricing and trade so as to drive prices towards their intrinsic value. This is unless there are sufficient other market players to offset the price inefficiencies (Grossman and Stiglitz 1980). ETF investors do not consider the fundamentals of the individual companies in the index (Hanaeda and Serita 2017; Sushko and Turner 2018) and, consequently, the prices of all stocks in the basket are likely to be inflated (deflated) by excess demand (supply) irrespective of whether such price movements are justified or not (Zou 2019). Empirical studies confirm that an increase in passive investing distorts market prices (Belasco et al. 2012; Zou 2019). The BOJ ETF purchases may thus impede the price discovery process as no fundamental analysis is conducted and the BOJ predominantly purchases when the market is in a downward swing (Harada and Okimoto 2021). Chen et al. (2019) present evidence that Japanese stocks with higher levels of BOJ ownership exhibit lower levels of price informativeness.

In this study, we conduct a comprehensive analysis of the impact of the BOJ’s ETF purchases on the efficiency of the Japanese market, considering both the TOPIX and Nikkei 225 indices. Efficiency is quantified using the Hurst exponent, which captures long-range dependence in prices, and the price delay metrics of Hou and Moskowitz (2005), which measure the speed with which new information is incorporated into stock prices. Using adjusted ordinary and feasible least squares to account for the estimated nature of the dependent variable, and controlling for other determinants of market efficiency, we find that an increase in ETF purchases by the BOJ results in lower long-range dependence for both indices while the impact on the price delay varies according to index and measure. Using the Bai and Perron (2003) breakpoint test, we further observe that the impact of the BOJ’s ETF purchases on market efficiency varies over time, with the dominant pattern being a delayed harmful effect, followed by a positive impact and thereafter a negative effect.

Our study makes several important contributions to the literature. First, we expand the nascent literature on the study of the BOJ’s unconventional monetary policy on stock markets. While studies have examined the impact on individual stock prices, liquidity, and capital structure (Hanaeda and Serita 2017; Barbon and Gianinazzi 2019; Harada and Okimoto 2021), to the best of our knowledge, only Chen et al. (2019), have explicitly examined the impact on market efficiency. We build on their study by considering fluctuations in efficiency on a daily rather than a quarterly basis and using a longer time period that includes the accelerated ETF purchases during the COVID-19 crisis. We also focus on efficiency at the market level rather than at the individual stock level, in line with Samuelson’s (1998) dictum that market efficiency may differ at the individual stock level compared to the market level. Second, our analysis sheds light on the impact of increased passive investment, as several studies have shown that increased passive investment contributes to market inefficiency (Belasco et al. 2012; Israeli et al. 2017; Zou 2019). Third, we follow the work of Coles et al. (2022) by considering two different aspects of efficiency—long-range dependence and speed of adjustment—which are often examined separately in studies (Al-Yahyaee et al. 2020; Köchling et al.^ 2019^) but rarely together. Fourth, drawing from the idea of time-varying efficiency under the AMH, our study contributes to further understanding what factors contribute to variation in market efficiency over time. Allied to this, we also build on prior studies of the efficiency of the Japanese market under the AMH (Noda 2016; Jiang and Li 2020) and EMH (Efremidze et al. 2021).

Inefficiency reduces the allocative efficiency of the stock market which lowers economic growth and returns, and triggers heightened volatility (Woolley and Bird 2003). As such, understanding the impact of the BOJ’s purchases on market efficiency is important as the BOJ considers the future of its ETF purchase programme and for other central banks that seek mechanisms to spur economic activity. Our study also provides insights as to the impact of increased passive investment management on market dynamics, which is important given the growing trend towards this management approach.

The remainder of this study is organised as follows: Section "Literature review" provides a brief review of the literature on the impact of increased passive management on market efficiency and the effect of the BOJ’s ETF purchases on the Japanese market. Section "Data and method" describes the data and method employed in this study. Section "Results" presents the results and Section "Conclusion" concludes.

## Literature review

Lorie and Hamilton (1973) were the first to suggest that increased passive investing will reduce competition for information in the market and so harm market efficiency. With the rise in passive investing in the last decade, several theoretical models have been developed to understand the consequence of passive investing on market efficiency. The model of Breugem and Buss (2019) infers that an increase in passive investing results in a decline in the number of shares that are price sensitive to new information, resulting in a decrease in informational efficiency. Similarly, Bond and Garcia (2022) conjecture that increased passive ownership contributes to a decline in aggregate market efficiency, meaning that market prices are more divorced from cash flows. However, through the welfare consequences of the shift towards passive investing, the relative efficiency of individual stocks increases. This is consistent with Samuelson’s (1998) dictum that markets can be micro-efficient but macro-inefficient. Gârleanu and Pedersen (2018) propose an equilibrium model of the optimal active and passive portfolios that illustrates that a decrease in the cost of passive investing, a result of increased fund flows to passive investments, results in a decrease in market efficiency. Therefore, these models predominantly conjecture a decline in market efficiency arising from greater passive investment.

Evidence largely supports the assertions of these theoretical models. Israeli et al. (2017) find that increased ETF ownership has contributed to a decline in informational efficiency as stocks move more in line with their sector and the broader market and less in line with their own earnings. Similarly, both Belasco et al. (2012) and Zou (2019) find that flows into passive funds cause an increase in the price-to-fundamental ratios of the underlying stocks. The results of Coles et al. (2022) reveal that the prices of stocks with a greater proportion of passive investors deviate more from a random walk, a measure of weak-form market efficiency. However, they find that price delay and the earnings response coefficient are not impacted by the proportion of passive investors. In reconciling these disparate results, Coles et al. (2022) argue that while increased passive investment does impact the price formation process, it does not affect the ability of arbitrageurs to trade and impound information into stock prices. Glosten et al. (2021) also identify that an increase in ETF ownership results in improved incorporation of earnings information into prices in the short run, but only for firms with a weak information environment.

Several studies have examined the effects of the BOJ’s ETF purchases on stock prices, liquidity and volatility. Results suggest prices of stocks included in the TOPIX and Nikkei 225 indices have increased more compared to those stocks not part of these indices. There is mixed evidence, however, as to whether the effects are temporary or sustained. Findings also suggest that the price impact has become smaller over time despite the increased purchase amounts by the BOJ (see Barbon and Gianinazzi 2019; Charoenwong et al. 2019; Harada and Okimoto 2021; Aono et al. 2021; Shen et al. 2021). Chen et al. (2019) examine whether holdings by the BOJ impact price efficiency at the stock level. They find that increased holdings by the BOJ results in lower price informativeness, as measured by variations from a random walk and price delay. Chen et al. (2019) also show that increased holdings by the BOJ lowered levels of market liquidity. With respect to volatility, Hanaeda and Serita (2017) observe that purchases of ETFs by the BOJ initially increased the return volatility of the constituent stocks but thereafter volatility declined. The impact of the BOJ’s purchases on company fundamentals, including capital structure, profits and investment, has also been examined (see Charoenwong et al. 2019; Gunji et al. 2021; Linh 2021).

Studying the efficiency of the Japanese market, Nagayasu (2003) documents evidence of long-range dependence in stock returns in violation of the weak-form EMH. Efremidze et al. (2021) also observe evidence against the weak-form EMH. However, their results reveal that the BOJ’s ETF purchases may have, at least temporarily, made markets more weak-form efficient. Noda (2016) finds that the efficiency of the TOPIX has varied over time, consistent with the AMH. Jiang and Li (2020) confirm that the TOPIX is weak-form efficient in normal conditions, but not in bull and bear markets.

Several key findings emerge from this literature. Across markets, there is evidence that increased passive investing has affected market efficiency. Studies on the BOJ’s unique ETF purchasing programme show that it has resulted in increases in the prices of the constituents of the TOPIX and Nikkei 225 indices, with some evidence that this policy has distorted the informativeness of stock prices. We build on this research framework by exploring the extent to which the BOJ’s ETF purchases have impacted the time-varying efficiency of the Japanese market.

## Data and method

### Data

The first part of our analysis is exploratory to compare the measures of market efficiency in the pre-BOJ ETF purchase period to those in the BOJ ETF purchase period. Thereafter, we set out to examine the impact of the BOJ’s ETF purchases on market efficiency after the introduction of the purchasing programme.

The BOJ targets its ETF purchases on the TOPIX and Nikkei 225 indices.^5^ The TOPIX comprises the first section of the TSE, tracking approximately 2000 stocks, and the Nikkei 225 comprises 225 stocks from the TSE first section. The TOPIX and Nikkei 225 are market capitalisation- and price-weighted indices respectively. The BOJ’s purchases of ETFs commenced in December 2010 although purchases accelerated substantially after the introduction of the Quantitative and Qualitative Easing (QQE) policy in April 2013 (Harada and Okimoto 2021). Closing price data for all variables (except where noted) are obtained from Bloomberg for all trading days. Returns are defined as the natural logarithmic differences in index levels multiplied by 100. We collect data from 1 January 2001 to 26 November 2021. Due to the use of rolling windows (see Section "Methods"), the pre-BOJ ETF purchase period is designated 20 January 2003 to 14 December 2010 and the BOJ ETF purchase period from 15 December 2010 to 17 November 2020. Both periods are used for the comparative analysis while the regression analysis is estimated for the BOJ ETF purchase period.

### Methods

We follow the extant literature (see for example, Cajueiro and Tabak 2004a, b; Rejichi and Aloui 2012; Hiremath and Narayan 2016) by calculating a rolling Hurst Exponent to measure the long-range dependence in the indices. A fixed window length of 500 observations is used. The Hurst exponent is estimated using the rescaled range, denoted $R/S$, which is a measure of the variability of a time series (Cajueiro and Tabak 2004a, b). The $R/S$ statistic is the range of partial sums of deviations of the return series from its mean ($R$), rescaled by its standard deviation ($S$). Mathematically, this is given by:

1 $${R/S}_{\tau }\equiv \frac{1}{{\widehat{\sigma }}_{\tau }} \left[\underset{1\le t\le \tau }{\mathrm{max}}\sum_{t=1}^{\tau }\left(r\left(t\right)-{\overline{r} }_{\tau }\right)-\underset{1\le t\le \tau }{\mathrm{min}}\sum_{t=1}^{\tau }\left(r\left(t\right)-{\overline{r} }_{\tau }\right)\right]$$

where $\{r\left(1\right), r\left(2\right)\dots r\left(\tau \right)\}$ is series of continuously compounded daily returns and ${\overline{r} }_{\tau }$ and ${\widehat{\sigma }}_{\tau }$ are the sample mean and standard deviation of $r\left(\tau \right)$ (Lo 1991). $R/S$ is described by the following empirical relationship:

2 $${R/S}_{\tau }\approx {C\tau }^{H}$$

where $C$ is a constant independent of $\tau$ and $H$ is the Hurst exponent.

If markets exhibit high levels of long-range dependence between return values, this implies that the movement of stock prices is not random, thus contradicting the weak-form EMH. If $0.5 < H < 1.0$, returns are positively autocorrelated meaning returns exhibit persistence. If $0 < H < 0.5$, returns are negatively autocorrelated meaning returns exhibit anti-persistence. If $H$ is approximately equal to 0.5, returns follow a random walk (returns are independent) or returns have only short-range dependence (Barunik and Kristoufek 2010). To filter the stock returns of any short-run dependence and thus ensure that if $H$ is approximately equal to 0.5 then the series can be considered to follow a random walk, the $R/S$ statistic is computed on the standardised residuals of an AR(1)-GARCH(1,1) model^6^ (Cajueiro and Tabak 2004a, b; Rejichi and Aloui 2012).^7^

The efficiency of the market has also been widely quantified using the price delay measures of Hou and Moskowitz (2005) which capture the speed with which prices reflect new information (see Hooy and Lim 2013; Bramante et al. 2015; Köchling et al. 2019). The greater the efficiency of the market, the smaller the price delay. We estimate a regression of the daily^8^ returns of each index against contemporaneous and lagged market returns as follows:

3 $${r}_{i,t}= {\alpha }_{i}+ {\beta }_{i}{r}_{m,t}+\sum_{k=1}^{2}{\delta }_{i,k}{r}_{m,t-k}+{\varepsilon }_{i,t}$$

where ${r}_{i,t}$ and ${r}_{m,t}$ are the returns on the index and market respectively and $k$ is the number of lags. The index is either the TOPIX or Nikkei 225. For the market index, we follow Lim and Hooy (2010) and Hooy and Lim (2013) by using an international index to capture the extent to which the Japanese indices respond to new global information, namely the MSCI All Country World Index excluding Japan (MSCI ACWI ex Japan).^9^ If the Japanese market responds immediately to new information, ${\beta }_{i}$ will be significantly different from zero and none of the ${\delta }_{i,k}$ coefficients will differ from zero (Hou and Moskowitz 2005). If there is a delayed market response, then some of the ${\delta }_{i,k}$ coefficients will differ from zero. Two lags of the market index are found to be optimal.

Using the estimates from this regression, we calculate two measures of price delay (Hou and Moskowitz 2005).^10^ The first measure, D1, is computed as one minus the ratio of ${R}^{2}$ from Eq. (3) with the restriction that ${\delta }_{i,k}=0, \forall k \in [\mathrm{1,2}]$ and the original ${R}^{2}$ from Eq. (3):

4 $$D1=1- \frac{{R}_{{\delta }_{i,k}=0, \forall k \in [\mathrm{1,2}]}^{2}}{{R}^{2}}$$

As longer lags are more severe for the efficiency of the market than shorter lags, a second delay measure is computed as follows:

5 $$D2= \frac{\sum_{k=1}^{2}k{\delta }_{i,k}}{{\beta }_{i}+\sum_{k=1}^{2}k{\delta }_{i,k}}$$

The regressions are estimated using a rolling window to allow for variation in the delay measure over time (Bramante et al. 2015). A window of 500 observations is again used.

To assess the impact of the BOJ’s purchases of passive ETFs on Japanese market efficiency, we build on the studies that have examined the determinants of long-range dependence (Hiremath and Narayan 2016; Al-Yahyaee et al. 2020) and price delay (Hooy and Lim 2013; Köchling et al. 2019). We estimate the following equation for the TOPIX and Nikkei 225 indices:

6 $${y}_{t}=\alpha + \theta {x}_{t}+\eta {z}_{t}+{\gamma d}_{t}+{e}_{t}$$

where ${y}_{t}$ is the measure of the efficiency of the index; ${x}_{t}$ refers to the value of the BOJ’s purchases of ETFs tracking the index; ${z}_{t}$ is a set of control variables; ${d}_{t}$ is an event dummy; and ${e}_{t}$ is the random error term. The measurement of the variables is outlined in Table 1.

**Table 1:** Measurement of the variables for each index

Variables	Acronym	Measure
Dependent variable		
Efficiency gap	EG	The Hurst exponent less 0.5.
Price delay	D1	The price delay measures of Hou and Moskowitz (2005).
	D2	
Independent variables		
BOJ ETF purchases	PUR	The total purchases by the BOJ of ETFs tracking the index.
ETF inflows	IF	The inflows to ETFs tracking the index.
Turnover ratio	TO	The number of trades per share in the index divided by the number of shares outstanding of all constituents of the index.
Volatility	VOL	The 30-day rolling standard deviation of the returns of the index.
Change in size	∆SIZE	The change in the natural log of the market capitalisation of all the constituents in the index.
Fukushima disaster	D_FD_	A dummy equal to one from 11 March to 11 April 2011 and zero otherwise.

To capture long-range dependence, we compute the deviation from efficiency using the Hurst exponent, termed the efficiency gap. A value close to zero indicates efficiency whereas a value close to 0.5 denotes inefficiency. This measure does not consider the possibility of anti-persistence (Vidal-Tomás 2022). A lower value thus indicates a more efficient market. For the price delay measures (D1 and D2), the lower the price delay, the more informationally efficient the market.

Data on the key explanatory variable, ETF purchases by the BOJ, was obtained from the BOJ website. This data reflects total purchases and does not distinguish between purchases of ETFs tracking the various indices. We use the weightings outlined by the BOJ to proportion the purchases per index over time.^11^ To ensure the robustness of the results, we compute a second measure of ETF purchases by the BOJ for each index, consisting of the daily net inflows to the ETFs tracking each of the indices (see Barbon and Gianinazzi 2019). If the BOJ’s purchases of ETFs contribute to a decline (improvement) in market efficiency, then the coefficient $\theta$ in Eq. (6) will be positive (negative).

Several control variables are specified. Liquidity, which we measure by the turnover ratio (TO), has been shown to attract arbitrage trading which contributes to increased efficiency (Chordia et al. 2008; Chung and Hrazdil 2010; Al-Yahyaee et al. 2020). Hence, a negative coefficient is expected. Arshad and Rizvi (2015) and Al-Yahyaee et al. (2020) illustrate that increased volatility hampers market efficiency. We thus expect a positive relationship between volatility and the efficiency measures. Finally, market size is included as size has been found to affect the level of efficiency, with larger markets exhibiting greater efficiency (Hooy and Lim 2013; Arshad et al. 2016). A negative coefficient is thus expected. A dummy variable is included for Japan’s 2011 Fukushima nuclear disaster, denoted from 11 March to 11 April 2011 (see Betzer et al. 2013).

The various efficiency measures are based on estimates and hence exhibit sampling variance. Employing ordinary least squares (OLS) to estimate Eq. (6) may thus lead to inefficient estimates. Accordingly, following Lewis and Linzer (2005), we use OLS adjusted for heteroscedasticity based on White’s standard errors and feasible generalised least squares (GLS) to ensure reliable results.

## Results

Table 2 presents the descriptive statistics for the efficiency measures in the pre-BOJ ETF purchase period, 20 January 2003 to 14 December 2010, and the BOJ ETF purchase period, 15 December 2010 to 17 November 2020. The efficiency gap of each index is close to zero in both periods suggesting low long-range dependence consistent with the weak-form EMH (Cajueiro and Tabak 2004a). For both the TOPIX and Nikkei 225, the mean efficiency gaps (0.0557 and 0.0558, respectively) are lower in the BOJ ETF purchase period than in the pre-BOJ purchase period (0.0596 and 0.0634, respectively). Similarly, the D1 price delay measures are lower in the BOJ ETF purchase period than in the prior period (for the TOPIX of 0.5799 and 0.5956, and for the Nikkei 225 of 0.5662 and 0.5809 for the respective periods). Contrastingly, the average measures for D2 are substantially higher in the BOJ ETF purchase period for the TOPIX and Nikkei 225 indices (0.5743 and 0.5099, respectively) compared to the prior period (0.4238 and 0.4267, respectively). D2 gives greater weightings to longer time delays than D1 (see Section "Methods") and thus the results suggest that information from two days prior (two lags were found to be optimal) takes longer to be incorporated into prices since 2010.

**Table 2:** Descriptive statistics for the variables before and during the BOJ ETF purchasing period

	TO	∆SIZE	VOL	EG	D1	D2	TO	∆SIZE	VOL	EG	D1	D2	PUR	IF
	*Panel A: TOPIX Pre-BOJ ETF Purchase Period*						*Panel B: TOPIX BOJ ETF Purchase Period*							
Mean	6649.35	− 2.42E−05	20.8981	0.0596	0.5956	0.4238	7176.87	0.0003	18.3970	0.0557	0.5799	0.5743	91.60	9584.91
Median	6671.13	0.0006	18.5300	0.0612	0.6225	0.4163	6727.14	0.0006	16.0700	0.0508	0.5844	0.5657	0.0000	48.6025
Std Dev	2268.19	0.0154	11.1715	0.0423	0.0982	0.0635	2822.99	0.0126	8.2780	0.0458	0.1114	0.0716	202.23	47,967.56
Kurtosis	0.1681	17.1415	15.6025	− 0.6288	− 0.9203	0.1666	5.7978	6.0266	2.6345	− 0.1902	− 0.2472	− 0.1108	8.8698	47.90
Skewness	0.3686	− 1.2161	3.3029	− 0.2355	− 0.6129	0.4711	1.7928	−0.4895	1.5668	0.4947	− 0.3906	0.4425	2.7106	2.6542
Min	1989.72	− 0.1886	6.4700	− 0.0676	0.3190	0.2417	2770.6156	−0.0997	5.9400	− 0.0475	0.2813	0.4321	0.0000	− 486,425.70
Max	17,537.84	0.1271	91.9700	0.1590	0.7965	0.6091	28,899.81	0.0758	49.0600	0.1794	0.8253	0.8354	1645.55	751,359.85
SW	0.9820***	0.8962***	0.7122***	0.9803***	0.8981***	0.9723***	0.8782***	0.9382***	0.8622***	0.9727***	0.9679***	0.9818***	0.5186***	0.5457

	*Panel C: Nikkei 225 Pre-BOJ ETF Purchase Period*						*Panel D: Nikkei 225 BOJ ETF Purchase Period*							
Mean	67704.27	1.44E−05	22.7422	0.0634	0.5809	0.4267	86414.91	0.0003	19.8580	0.0558	0.5662	0.5099	33.06	3044.67
Median	66693.44	0.0006	20.7650	0.0662	0.5932	0.4134	81210.76	0.0005	17.3850	0.0488	0.5741	0.5158	0.0000	986.15
Std Dev	26266.44	0.0162	12.4975	0.0453	0.0832	0.0670	35996.94	0.0131	8.7445	0.0536	0.1060	0.0787	61.54	39730.90
Kurtosis	− 0.0585	9.0717	17.1003	− 0.3490	− 0.8700	1.7802	7.1758	5.4931	2.5893	− 0.6963	− 0.0515	−0.7688	4.4882	38.02
Skewness	0.4754	− 0.5416	3.5145	− 0.3968	− 0.2532	− 0.0668	1.9118	−0.3691	1.5292	0.2859	− 0.5628	−0.2310	2.0155	− 1.4211
Min	15,462.26	− 0.1363	7.5800	− 0.0629	0.3234	0.1947	24397.14	−0.1004	6.8200	− 0.0559	0.2619	0.3195	0.0000	− 533,748.28
Max	183,521.16	0.1377	104.0400	0.1585	0.7480	0.6540	388747.51	0.0802	55.2500	0.1835	0.7977	0.7191	388.49	351,848.71
SW	0.9741***	0.9200***	0.6857***	0.9793***	0.9604***	0.9311***	0.8729***	0.9438***	0.8700***	0.9769***	0.9541***	0.9780***	0.6013***	0.6606***

This table presents the descriptive statistics for the variables in the pre-BOJ ETF purchase period (20 January 2003–14 December 2010) and during the BOJ ETF purchase period (15 December 2010–17 November 2020). TO refers to the turnover of the index, ∆SIZE refers to the change in the natural logarithm of the market capitalisation of the index, VOL refers to the 30-day rolling return volatility of the index, EG refers to the efficiency gap (defined as H—0.5, where H is the Hurst exponent), D1 and D2 refer to the two price delay measures of Hou and Moskowitz (2005), PUR refers to the value of the BOJ’s ETF purchases and IF refers to the net inflows to ETFs tracking the index. SW is the Shapiro Wilk test of normality. ***indicates significance at 1%

**Fig. 1:**
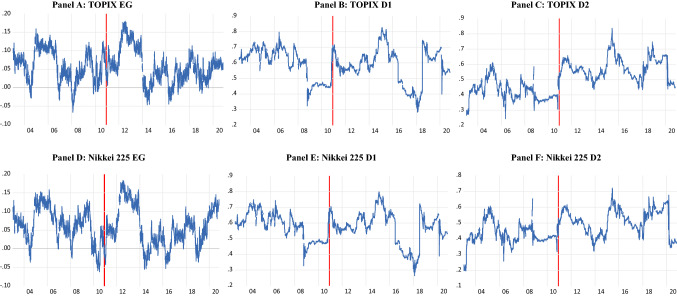
Efficiency measures for the TOPIX and Nikkei 225 indices. This figure plots the efficiency measures for the TOPIX (**A**–**C**) and Nikkei 225 (**D**–**F**). EG is the Hurst exponent less 0.5 and D1 and D2 are the first and second price delay measures of Hou and Moskowitz (2005). The red vertical line on 15 December 2010 denotes the commencement of the BOJ’s ETF purchase programme

Figure 1 plots the efficiency measures for the two indices from 2003. The red vertical line on 15 December 2010 denotes the commencement of the BOJ’s ETF purchase programme. The graphs demonstrate that both long-range dependence and price delay vary substantially over time. This supports the assertions of the AMH of time-varying efficiency, consistent with the findings of Noda (2016) and Jiang and Li (2020) for the Japanese market. From 2012, the efficiency gaps of both indices experience a sharp rise, suggesting greater dependency in prices. This increase dissipates quite quickly with the efficiency gaps reaching notable lows in early 2014. The improvement in efficiency could be attributable to the greater ETF purchases by the BOJ from April 2013 under the QQE policy. This effect would be somewhat delayed because of the rolling window of historical observations used to estimate the Hurst exponent. From this point onwards, the long-range dependency continues to exhibit upward and downward movements but what is noticeable is that the high points are lower than previously. This is consistent with the finding that the average efficiency gap for both the TOPIX and Nikkei 225 are lower in the BOJ period (Table 2).

D1 exhibits no sharp movements until early 2014 when a dramatic spike occurs for both indices (Fig. 1). The price delay measures also rely on a rolling window and thus this spike may be attributable to the acceleration of ETF purchases by the BOJ in April 2013. This rise in D1 persists only briefly before commencing a decline which continues until early 2018 before another sharp rise. D1 predominantly fluctuates in the range 0.6–0.7 for the TOPIX and 0.55—0.65 for the Nikkei 225 thereafter. An increase in D2 also occurs from 2014 onwards. However, when the value of D2 declines, it does not decrease as far as seen previously and remains above previous lows as it fluctuates over time. This confirms the conclusion from Table 2 that D2 for both the TOPIX and Nikkei 225 is substantially higher in the BOJ ETF purchase period compared to the prior period. It is thus evident that the long-range dependence and price delay have changed since the introduction of the BOJ’s ETF purchase programme. Whether these changes can be attributed to the BOJ’s monetary policy is investigated further below.

**Fig. 2:**
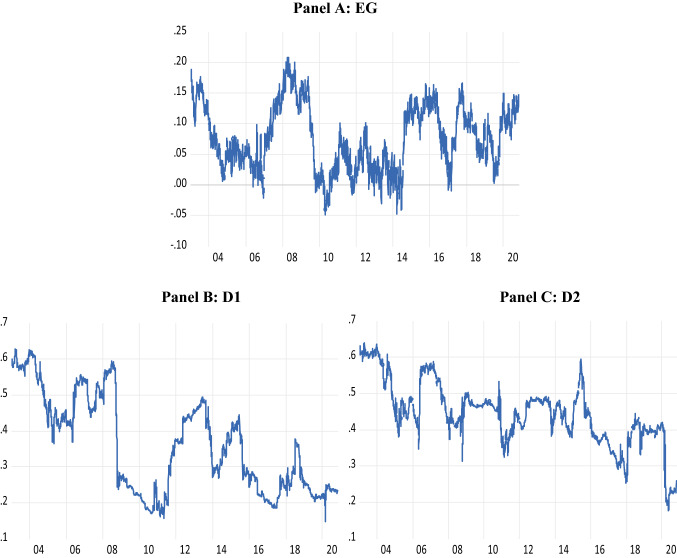
Efficiency measures for the MSCI Asia ex Japan index. This figure plots the efficiency measures for the MSCI Asia ex Japan index. EG is the Hurst exponent less 0.5 (**A**) and D1 and D2 are the first and second price delay measures of Hou and Moskowitz (2005) (**B** and **C**, respectively)

As a comparative analysis, we examine the efficiency of the MSCI Asia excluding Japan index (MSCI Asia ex Japan).^12^ Figure 2 shows that this index also exhibits time-varying long-range dependence and price delay. D1 and D2 exhibit several peaks and troughs but, overall, there is a decline in price delay over the period, i.e., faster incorporation of new information across Asian markets. This contrasts with the trend for the Japanese indices seen in Figure 1 of increasing price delay post 2010. The efficiency gap of the Asia ex Japan index is lower from 2010 to 2014 than from 2003 to 2009. Thereafter, long-range dependence increases notably although wide fluctuations are evident. In comparison to the Japanese market, the lower efficiency gap of Asian markets occurs earlier than seen in Japan and is more persistent. Thus, the improvement in long-range dependence seen in Figure 1 for Japan may not necessarily be entirely due to the BOJ’s purchase programme but also due to other events/news which impacted Asia’s markets as well such as the 2013 taper tantrum^13^ and the bursting of China’s stock market bubble and economic downturn in 2015 which raised fears that it would spark another financial crisis (Shu et al. 2015; Kaletsky 2015). Similarly, changing efficiency in 2018 across the Japanese and Asian indices may reflect events such as the launch of missiles by North Korea and the Chinese government’s regulation of internet finance companies during 2017 (Mundey 2017). The fact that the timing differs between Asian and Japanese markets, however, may reflect the role of the BOJ’s purchase programme.

As all variables are stationary based on the Augmented Dickey-Fuller test, we proceed to estimate Eq. (6).^14^ The results for OLS with White’s standard errors are shown in Table 3. The findings using FLS (Table 6 in the “Appendix”) confirm those obtained using OLS. The BOJ’s ETF purchases have a significant negative impact at the 1% level on both the TOPIX and Nikkei 225 efficiency gap, with the impact larger for the latter (coefficients of −1.14E−05 and −0.0002, respectively). This suggests that an increase in ETF purchases by the BOJ contributes to an improvement in efficiency due to lower long-range dependence. This follows the findings of Efremidze et al. (2021) that the BOJ purchase programme may have temporarily improved weak-form efficiency of the Japanese market. Net ETF inflows have no impact on the efficiency gap.

**Table 3:** Results from the adjusted OLS regressions

Dependent variable	EG	EG	D1	D1	D2	D2
	*Panel A: TOPIX*					
Intercept	0.0335***	0.0321***	0.5345***	0.5255***	0.5913***	0.5884***
PUR	− 1.14E−05***		− 7.44E−05***		− 2.35E−05***	
IF		− 1.84E−08		− 1.41E−07***		− 4.48E−08
∆SIZE	−0.0242	0.0389	− 0.4202***	− 0.0049	− 0.3444***	− 0.2133*
TO	3.62E−06***	3.74E−06***	3.17E−06***	3.94E−06***	− 2.97E−06***	− 2.73E−06***
VOL	−0.0002	− 0.0002*	0.0016***	0.0015***	0.0003*	0.0003
D_FD_	0.0032	0.0040	− 0.0342***	− 0.0293***	0.0370***	0.0385***
Adjusted* R*^2^	0.0492	0.0511	0.0303	0.0425	0.0120	0.0149
	*Panel B: Nikkei 225*					
Intercept	0.0419***	0.0394***	0.4885***	0.4881***	0.5424***	0.5438***
PUR	− 0.0002***		− 2.68E−05		9.13E−05***	
IF		− 3.08E−08		− 4.84E−08		2.49E−08
∆SIZE	− 0.2850***	0.0505	− 0.0447	0.0179	− 0.0682	− 0.2599**
TO	1.88E−07***	1.65E−07***	5.98E−07***	5.94E−07***	− 2.64E−07***	− 2.51E−07***
VOL	0.0002	0.0001	0.0014***	0.0014***	− 0.0007***	− 6.48E−04***
D_FD_	− 0.0127***	− 0.0073*	− 0.0252***	− 0.0245***	0.0856***	0.0826***
Adjusted * R*^2^	0.0124	0.0390	0.0665	0.0663	0.0271	0.0310

This table presents the results of the regressions of the BOJ’s ETF purchases against various measures of market efficiency using OLS with White’s heteroscedasticity-consistent standard errors. Panels A and B present the results for the TOPIX and Nikkei 225 respectively. TO refers to the turnover of the index, ∆SIZE refers to the change in the natural logarithm of the market capitalisation of the index, VOL refers to the 30-day rolling return volatility of the index, EG refers to the efficiency gap (defined as H—0.5, where H is the Hurst exponent), D1 and D2 refer to the two price delay measures of Hou and Moskowitz (2005), PUR refers to the value of the BOJ’s ETF purchases and IF refers to the net inflows to ETFs tracking the index. ***, ** and * indicate significance at 1%, 5% and 10%, respectively

Differences are observed across the indices for the price delay measures. The BOJ’s ETF purchases have a significant negative impact on D1 for TOPIX but not the Nikkei 225 (−7.44E−05 and −2.68E−05, respectively). The same is seen with net ETF inflows (−1.41E−07 and −4.84E−08, respectively). The BOJ’s ETF purchases have a negative impact on D2 of the TOPIX (−2.35E−05) but a positive impact for the Nikkei 225 (9.13E−05). ETF inflows have no significant impact on D2 for both indices. The negative sign on the BOJ’s ETF purchases for the TOPIX suggests that the BOJ’s purchasing programme decreases the price delay meaning that information is incorporated into prices faster. In contrast, for the Nikkei 225, the purchasing programme results in an increase in the price delay when longer delays are given greater weighting (D2 vs. D1).

This difference across indices could be attributable to the different weightings of the TOPIX and Nikkei 225 indices. The Nikkei 225 index is price weighted whereas the TOPIX is market capitalisation weighted. According to the EMH, fundamental information should be based on market capitalisation and, as such, the allocation of funds by the BOJ based on non-market weights may exacerbate the negative effects on market efficiency (Barbon and Gianinazzi 2019). To investigate the impact of index weighting, we created a synthetic Nikkei 225 index, accounting for all index additions/ deletions over the period, where the constituents are weighted according to market capitalisation rather than price.^15^ Results using adjusted OLS are shown in Table 4. Consistent with the findings for both the TOPIX and Nikkei 225 indices, the BOJ’s purchases contribute to a decline in the efficiency gap of the synthetic index. The BOJ’s purchases have no impact on D1 but a positive and significant impact on D2. The significant positive impact on D2 resembles that seen for the canonical Nikkei 225. ETF inflows have no impact on the efficiency measures of the synthetic index, as with the Nikkei 225. This analysis suggests that the different weighting mechanisms do not appear to directly account for the differing findings on the TOPIX relative to the Nikkei 225. Differing levels of liquidity between stocks in the Nikkei 225 versus the TOPIX may be a contributing factor. The Nikkei 225 selects stocks which are highly liquid (Chen et al. 2019). Thus, more liquid stocks may be negatively impacted by the trades of the BOJ whereas less liquid stocks (such as some of those in the TOPIX) are positively impacted (see also Chen et al. 2019). We recommend the analysis of liquidity of the index constituents as an avenue for further research.

**Table 4:** Results for the synthetic Nikkei 225

Dependent variable	EG	EG	D1	D1	D2	D2
Intercept	0.0234***	0.0212***	0.5200***	0.5194***	0.5724***	
PUR	− 0.0001***		− 3.50E−05		9.55E−05***	
IF		− 2.51E−08		− 6.44E−08		1.71E−08
∆Size	− 0.2295***	0.0794	− 0.0728	0.0118	− 0.0012	− 0.2066*
TO	3.14E−07***	2.93E−07***	4.4E−07***	4.36E−07***	− 1.1E−08	2.96E−09
VOL	0.0002*	0.0002	0.0014***	0.0014***	− 0.0004*	− 0.0004*
*D*_FD_	− 0.0136***	− 0.0086**	− 0.0371***	− 0.0362***	0.0377	0.0344***
Adjusted * R*^2^	0.0758	0.0499	0.0382	0.0385	0.0073***	0.0022

This table presents the results of the regressions of the BOJ’s ETF purchases against various measures of market efficiency using OLS with White’s heteroscedasticity-consistent standard errors for the synthetic Nikkei 225 index, which weights the constituents of the Nikkei 225 according to market capitalisation rather than price. TO refers to the turnover of the index, ∆SIZE refers to the change in the natural logarithm of the market capitalisation of the index, VOL refers to the 30-day rolling return volatility of the index, EG refers to the efficiency gap (defined as H—0.5, where H is the Hurst exponent), D1 and D2 refer to the two price delay measures of Hou and Moskowitz (2005), PUR refers to the value of the BOJ’s ETF purchases and IF refers to the net inflows to ETFs tracking the index. ***, ** and * indicate significance at 1%, 5% and 10%, respectively

The finding that the BOJ’s ETF purchasing programme has largely contributed to improved speed in the incorporation of information into prices and lower long-range dependence in prices differs from the result of Chen et al. (2019) of an adverse effect on the efficiency of Japanese stocks. The differences may reflect varying methodologies and time periods studied. Chen et al. (2019) examined efficiency at the firm level across quarters whereas our analysis focuses on indices daily. Also, their sample ended in 2016 where our sample extends to November 2021, capturing further acceleration in the ETF purchasing programme including that driven by the COVID-19 pandemic. The differences in findings are consistent with Samuelson’s (1998) dictum that market efficiency may vary at the firm level versus the stock market level.

Given the variability in the measures of market efficiency over time (Figure 1), it may be that the relationship between the BOJ’s ETF purchases, and the efficiency of the Japanese indices has varied. We therefore test for breakpoints in this relationship in Eq. (6) using the Bai and Perron (2003) test. The results are presented in Table 5. Four breakpoints in the relationship between the efficiency gap and the BOJ’s ETF purchases occur for both indices. ETF purchases have a significant impact on the efficiency gap across all periods, but the signs vary. The coefficient is positive for the first two subperiods; negative for the second two subperiods; and positive for the final subperiod. This suggests that initially the BOJ’s purchases resulted in a decline in efficiency but from approximately December 2013 efficiency improved before a fall again after 2018 and 2019 for the TOPIX and Nikkei 225, respectively. When net ETF inflows are used, three breakpoints are identified. Up until 2012/ 2013, the BOJ’s programme harms efficiency (a significant positive coefficient), followed by an improvement in efficiency (a significant negative coefficient) until the end of 2016/ 2017, while there is no impact in the final sub-period. Efremidze et al. (2021) also noted that the improvement in weak-form efficiency of the Japanese market due to the BOJ’s monetary policy may have been temporary. Similarly, Hanaeda and Serita (2017) showed that the impact of the purchasing programme on market volatility varied over time.

**Table 5:** Results from the OLS breakpoint regressions

Dependent variable	EG	EG	D1	D1	D2	D2
	*Panel A: TOPIX*					
Breaks	12/6/2012 17/12/2013 8/9/2016 5/3/2018	20/11/2012 10/10/2017	20/5/2014 14/6/2016 2/7/2018	30/6/2016 13/2/2018	19/3/2013 11/11/2014 6/3/2018	23/5/2019
Intercept	0.0355***	0.0315***	0.5530***	0.5317***	0.5927***	0.5905***
PUR	0.0003*** 0.0005*** − 0.0003*** − 3.95E−05*** 3.74E−07***		3.51E−05 0.0009*** − 0.0004*** 5.09E−05***		0.0001 − 0.0006*** 0.0007*** − 0.0001*** 2.52E−06	
IF		2.21E−06*** − 1.47E−07*** 1.69E−08		2.54E−07*** − 1.29E−06*** − 4.81E−09***		1.89E−08 − 5.43E−07***
∆SIZE	− 0.0580	0.0401	0.2576*	− 0.0186	− 0.1135	− 0.2113*
TO	3.67E−06***	3.87E−06***	2.85E−06***	4.12E−06***	− 2.64E−06***	− 3.35E−06***
VOL	− 0.0002***	− 0.0002**	0.0004*	0.0012***	1.60E−05	0.0005***
D_FD_	− 0.0026	0.0048	0.0061	− 0.0231*	0.0452***	0.0353***
Adjusted R^2^	0.1507	0.0682	0.2709	0.0739	0.1628	0.0269
	*Panel B: Nikkei 225*					
Breaks	12/6/2012 26/12/2013 5/8/2016 22/5/2019	13/112013 11/10/2016	20/5/2014 16/9/2016 2/7/2018	No breaks	18/2/2013 19/1/2015 12/7/2016 6/2/2018	5/11/2012 16/9/2014 23/3/2016
Intercept	0.0422***	0.0389***	0.5173***		0.5411***	0.5442***
PUR	0.0001** 0.0005*** − 0.0003*** − 1.14E−04*** 4.07E−04***		9.71E−05** 0.0003*** − 0.0015*** 2.16E−04***		0.0002*** − 0.0007*** 0.0004*** − 0.0001*** 1.74E−04**	
IF		9.57E−07*** − 4.35E−07*** 4.09E−09				8.18E−07*** − 1.01E−06*** 8.66E−07*** − 2.47E−08
∆SIZE	− 0.0553	0.0618	0.0927		− 0.0628	− 0.2614
TO	2.02E−07***	1.79E−07***	4.97E−07***		− 2.19E−07***	− 2.64E−07**
VOL	− 0.0001	0.0001	0.0005**		− 7.27E−04***	− 0.0006***
D_FD_	− 0.0106**	− 0.0072*	− 0.0031		0.0798***	0.0838***
Adjusted R^2^	0.1488	0.0349	0.2608		0.1417	0.0511***

This table presents the results of the regressions of the BOJ’s ETF purchases against various measures of market efficiency using OLS breakpoint regressions with White’s heteroscedasticity-consistent standard errors. The breakpoints are determined using the Bai and Perron (2003) test. Panels A and B present the results for the TOPIX and Nikkei 225 respectively. TO refers to the turnover of the index, ∆SIZE refers to the change in the natural logarithm of the market capitalisation of the index, VOL refers to the 30-day rolling return volatility of the index, EG refers to the efficiency gap (defined as 1—H, where H is the Hurst exponent), D1 and D2 refer to the two price delay measures of Hou and Moskowitz (2005), PUR refers to the value of the BOJ’s ETF purchases and IF refers to the net inflows to ETFs tracking the index. ***, ** and * indicate significance at 1%, 5% and 10%, respectively

For the price delay, an oscillating impact exists over subperiods. With the TOPIX, ETF purchases initially have no impact on D1 followed by a positive impact and then a negative impact. In contrast for the Nikkei 225, the impact is initially positive, then negative and then reverts to positive again. ETF net inflows show a similar positive, then negative effect on the Nikkei 225 but the negative effect remains thereafter. No breakpoints are identified for the Nikkei 225 when ETF purchases are used. For the TOPIX the impact on D2 is insignificant, negative, positive, negative and insignificant across the five subperiods respectively whereas for the Nikkei 225, the impact on D2 is positive, negative, positive negative and positive respectively. ETF net inflows have an insignificant effect followed by a negative impact on the TOPIX but an initial positive impact on the Nikkei 225 followed by a negative, positive and then insignificant impact. Overall, for D1 and D2, the results largely indicate that the BOJ ETF purchasing programme initially has no impact on the speed of information incorporation into prices of the TOPIX, but thereafter the purchases slow information incorporation before enhancing price informativeness. However, as seen with the efficiency gap, a harmful effect appears to materialise later in the sample period. For the Nikkei 225, the BOJ’s purchases immediately hinder price delay before then reducing price delay.

What emerges from the subperiod analysis is of a complex and dynamic relationship between the BOJ’s ETF purchases and market efficiency. There appears to be evidence that in the early years, the purchases harmed market efficiency, with both long-range dependence and price delay increasing. Thereafter, however, the ETF purchases contributed to an improvement in price efficiency. The BOJ purchases usually occur following a substantial negative return over the previous evening and morning market, especially when the return is below the third decile of the historical return distribution (Hattori and Yoshida 2020). This may reflect market participants that have overreacted to negative news driving prices too low. As such, the ETF purchases by the BOJ drive prices back to their intrinsic value thus acting counter-cyclically. However, from approximately 2018 onwards, a harmful effect of the BOJ’s purchases on efficiency is observed. This may reflect that by this point in time, the BOJ’s purchases reflected little fundamental information but were simply an attempt to stimulate the market, especially with the impact of COVID-19 (Lee and Fujioka 2022).

As noted in Figure 2, the efficiency gap and price delay measures of the MSCI Asia ex Japan index exhibited notable trend changes from 2014 to 2016, possibly driven by the taper tantrum (2013) and fears of an impending financial crisis due to China’s economic downturn and stock market crash (2015). The breakpoints observed in the relationship between ETF purchases by the BOJ and Japanese market efficiency in the period thereafter (late 2015 to 2016) may also reflect these events (the rolling window accounting for the delayed impact). Likewise, the breakpoints witnessed in 2018 may reflect changing trading behaviour in light of repeated North Korean missiles as well as the Chinese government’s regulation of internet finance companies in 2017. The comparative analysis thus reveals that the impact of the BOJ’s purchases on Japanese market efficiency, which vary over time, may also be driven by external events beyond those in Japan only.

The results in Tables 3 and 4 show that turnover has a significant impact on the efficiency measures but with a positive sign for the efficiency gap and D1 but a negative sign for D2. The negative sign is consistent with expectations that increased turnover attracts more arbitrageurs and thus supports market efficiency (Chordia et al. 2008; Al-Yahyaee et al. 2020). However, Chordia et al. (2008) and Chung and Hrazdil (2010) also demonstrate that a negative relationship between liquidity and efficiency may arise due to adverse selection whereby those participants remaining in the market when liquidity is low, have greater incentives to gather more information and trade on this information. Chen et al. (2019) also observe varying signs for turnover across the efficiency measures in the Japanese market. The impact of volatility on market efficiency is mostly significant but the signs are inconsistent. Accordingly, this suggests that volatility does not have a persistent adverse effect on Japanese market efficiency (Arshad and Rizvi, 2015). Size has only limited impact on market efficiency but when significant, the coefficient is negative confirming that increased size results in an improvement in efficiency (Hooy and Lim 2013). The Fukushima nuclear disaster largely results in an increase in efficiency (as reflected by negative coefficients) except for D2.

## Conclusion

The BOJ’s unconventional monetary policy of purchasing ETFs was initially introduced as a temporary programme yet remains more than a decade later. Purchases rapidly accelerated from 2013 onwards but tapered off substantially in 2021. Several studies have examined the impact of these ETF purchases on stock returns, liquidity and volatility. However, very little attention has been given to the impact on efficiency. In this study, we investigate whether increased ETF purchases by the BOJ impact the efficiency of the TOPIX and Nikkei 225 indices.

Our findings show that the ETF purchase programme has enhanced weak-form market efficiency as measured by the efficiency gap across both the TOPIX and Nikkei 225 indices. The purchase programme has also contributed to improved speed of information incorporation for the TOPIX but not for the Nikkei 225 where the price delay worsens especially with longer delays. Results suggest that this difference is not due to the different weightings of the two indices. A breakpoint analysis reveals a time-varying impact of ETF purchases on the efficiency measures. The general trend that emerges is that the BOJ programme had no initial impact on market efficiency but thereafter hampered efficiency before improving efficiency although the timing thereof differs across indices and efficiency measures. But what also emerges is that in the latter years, the purchase programme has hindered market efficiency again.

These findings have important implications for other central banks that may be forced to directly intervene in stock markets in unprecedented times. Moreover, the findings of this study also have important lessons for the BOJ when they unwind their ETF holdings. For these policy makers our results show that large scale purchases impact market efficiency, both positively and negatively. Adopting a counter-cyclical policy of buying (selling) when prices are abnormally low (high) appears to better support market efficiency as it counteracts potential overreaction caused by market participants. Creative solutions could be implemented by the BOJ to minimise market disruptions due to the sale of ETFs. For example, Koll (2021) recommends encouraging retirees to purchase ETFs by making the ETFs exempt from inheritance tax in Japan, which is the highest in the world.

As shown in this study, a time-series analysis allows for a more granular approach and thus we suggest expanding on the work of this study and Chen et al. (2019) by looking at individual stocks using data of a daily frequency to further understand the impact of the BOJ’s ETF purchases on market efficiency.

## Appendix

See Table 6.

**Table 6:** Results from the FLS regressions

Dependent variable	EG	EG	D1	D1	D2	D2
	*Panel A: TOPIX*					
Weight	0.0394***	0.0408***	0.5310***	0.5246***	0.5890***	0.5858***
PUR	− 8.46E−06**		− 3.69E−05***		− 2.08E−05***	
IF		− 2.22E−08		− 1.09E−07***		− 6.87E−09
∆Size	− 0.0185	0.0043	− 0.1747*	0.0020	− 0.2051***	− 0.1475***
TO	2.30E−06***	2.44E−06***	4.41E−06***	5.22E−06***	− 3.22E−06***	− 2.89E−06***
VOL	− 1.85E−05	− 0.0002	0.0013***	0.0011***	0.0005***	0.0005***
*D*_FD_	0.0062	0.0101**	− 0.0351***	− 0.0326***	0.0360***	0.0375***
Adjusted R^2^	0.2461	0.2261	0.9327	0.9481	0.9698	0.9542
	*Panel B: Nikkei 225*					
Weight	0.0513***	0.0486***	0.5017***	0.4951***	0.5313***	0.5315***
PUR	− 0.0001***		− 3.03E−05		0.0001***	
IF		− 1.31E−08		− 2.46E−08		3.31E−08
∆Size	− 0.2420***	− 0.0408	− 0.0711	− 0.0127	− 0.0661	− 0.1685***
TO	3.90E−08	7.56E−08**	5.67E−07***	6.53E−07***	− 3.09E−07***	− 2.90E−07***
VOL	0.0001	2.53E−05	0.0009***	0.0008***	1.39E−05	0.0002
*D*_FD_	0.0002	− 0.0009	− 0.0140*	− 0.0178**	0.0698***	0.0624***
Adjusted R^2^	0.1530	0.0767	0.8620	0.8972	0.9647	0.9477

This table presents the results of the regressions of the BOJ’s ETF purchases against various measures of market efficiency using FLS. Panels A and B present the results for the TOPIX and Nikkei 225 respectively. Weight is the weighting used under FLS. TO refers to the turnover of the index, ∆SIZE refers to the change in the natural logarithm of the market capitalisation of the index, VOL refers to the 30-day rolling return volatility of the index, EG refers to the efficiency gap (defined as H—0.5, where H is the Hurst exponent), D1 and D2 refer to the two price delay measures of Hou and Moskowitz (2005), PUR refers to the value of the BOJ’s ETF purchases and IF refers to the net inflows to ETFs tracking the index. ***, ** and * indicate significance at 1%, 5% and 10%, respectively

## Footnotes

1 For further details on the changes in the BOJ’s asset purchase programme see Hanaeda and Serita (2017) and Harada and Okimoto (2021). The ¥6 trillion limit was doubled in 2020 due to the Covid-19 pandemic.

2 The Hong Kong Monetary Authority purchased domestic stocks during the Asian Financial Crisis in 1998. Although these were large in magnitude, they only lasted two weeks (Hanaeda and Serita 2017).

3 In June 2020, the bank launched a lending scheme whereby it lends its ETF holdings to market participants, but the programme has struggled to attract investor interest.

4 Although some ETFs adopt a synthetic replication strategy, for the purposes of this study, physical replication is assumed given that ETFs purchased by the BOJ follow this approach.

5 The JPX-Nikkei 400 was introduced as a targeted index on 19 November 2014. However, purchases of ETFs tracking this index constituted only 4% of the BOJ's total ETF purchases prior to 2016. Hence, this index is not included in this study (see also Barbon and Gianinazzi 2019).

6 The conditional mean and variance equations for the AR(1)-GARCH(1,1) models are ${r}_{t}=\mu +{\gamma r}_{t-1}+{\varepsilon }_{t}$ and ${h}_{t}=\omega +\alpha {\varepsilon }_{t-1}^{2}+\beta {h}_{t-1}$, respectively. The standardised residuals are $\xi \left( t \right) = \varepsilon \left( t \right)/\sqrt {h\left( t \right)}$.

7 Although Lo (1991) proposed a modification to the $R/S$ statistic which is robust to short-run dependence, this modification is criticised as it only provides a conservative estimate of short-range dependence (Teverovsky et al. 1999).

8 Although weekly data is frequently used to estimate the price delay measure, using daily data results in more precise estimates (Hou and Moskowitz 2005; Bramante et al. 2015).

9 This index covers approximately 85% of the global equity opportunity set outside of Japan.

10 Hou and Moskowitz (2005) propose a third measure of price delay which adjusts for the precision of the estimates. This was estimated but due to its almost perfect correlation with D2 (0.99), results are not shown.

11 From December 2010 to 18 November 2014, 46% and 54% were allocated to ETFs tracking the TOPIX and Nikkei 225 respectively. Thereafter until 20 September 2016, 42% of purchases were directed at the TOPIX and 53% to the Nikkei 225. For the next period running to 30 July 2018, 49% of purchases were allocated to the TOPIX, and 50% split between the TOPIX and Nikkei 225. Until 19 March 2021, 73.68% of purchases were directed at TOPIX-tracking ETFs and the remaining 25.3% split between ETFs tracking the TOPIX and Nikkei 225. For the final period, only ETFs tracking the TOPIX were purchased (BOJ 2021).

12 This index comprises stocks from two developed and eight emerging markets in Asia. It was chosen given the findings of prior studies showing similarity in price delay across Asian markets (see Lim & Hooy 2010; Hooy & Lim 2013), thus allowing for a comparison to the efficiency of Japan’s market since the commencement of the BOJ’s ETF purchase programme. We thank an anonymous reviewer for the suggestion of a comparative analysis.

13 The taper tantrum refers to the response by markets to the unexpected news that the United States Federal Reserve was reducing its quantitative easing policy.

14 The results of the unit root tests are available from the authors upon request.

15 We would like to thank an anonymous reviewer for this valuable suggestion.
